# What Is the Pathway to the Best Model of Care for Traumatic Spinal Cord Injury? Evidence-Based Guidance

**DOI:** 10.46292/sci23-00059S

**Published:** 2023-11-17

**Authors:** Matheus Joner Wiest, Judith Gargaro, Mark T. Bayley, Neurotrauma Care Pathways Steering Committee, Alice Bellavance, Barry Munro, Bob Teasell, Cathy Craven, Charissa Levy, James Milligan, Lauren Fehlings, Laurie Tucker, Ruth Wilcock, Stephen Gregory, Stuart Howe

**Affiliations:** 1 KITE Research Institute, Toronto Rehabilitation Institute – University Health Network, Toronto, ON, Canada; 2 Division of Physical Medicine and Rehabilitation, University of Toronto, Toronto, ON, Canada

**Keywords:** care pathway, clinical practice guidelines, equity, integrated care, model of care, spinal cord injury

## Abstract

**Introduction:**

People with traumatic spinal cord injury (tSCI) experience lifelong physical and emotional health impacts, needing specialized care that is complex to navigate. The non-standardized care pathways used by different jurisdictions to address these needs lead to care inequities and poor health outcomes.

**Purpose:**

To develop an evidence-based integrated tSCI Care Pathway, from time of injury to life in the community.

**Methods and Analysis:**

Eighty key partners engaged in planning, providing, and receiving tSCI care (1) identified existing guidelines, pathways, and care models; (2) created the tSCI Care Pathway with key elements or building blocks (“the what”), not specific recommendations (“the how”) for each care stage (Acute, Rehabilitation, and Community), with elements highlighting the role of primary care and equity considerations on the pathway; (3) identified regional gaps in the tSCI Pathway and prioritized them for implementation; and (4) developed quality indicators.

**Outcomes:**

The tSCI Pathway was drafted in overarching and detailed formats. For Acute Care, building blocks focused on appropriate assessment, initial management, and transition planning; for Rehabilitation, building blocks focused on access to specialized rehabilitation and assessment and planning of community needs; for Community, building blocks focused on follow-up, mechanisms for re-access, and holistic support for persons and families; and for equity considerations, building blocks focused on those at-risk or requiring complex supports. Team-based primary care and navigation supports were seen as crucial to reduce inequities.

**Conclusion:**

This is the first comprehensive care pathway for tSCI. The Pathway is grounded in person-centred care, integrated care and services, and up-to-date clinical practice guidelines. The tSCI Care Pathway is flexible to regional realities and individual needs to ensure equitable care for all.

## Introduction

People with traumatic spinal cord injuries (tSCIs) require highly specialized care that addresses the lifelong impacts on their physical^1^ and cognitive^2^ health and their ability to participate in the community.^3^ Planning for appropriate care and supports is challenging because (1) injuries vary by type, severity, and associated secondary complications, and (2) variations in geographic location and payor (e.g., insurance, extended healthcare benefits) influence access to services. Healthcare needs after tSCI are also influenced by ageism^4^ and the social determinants of health^5^-^7^, resulting in great disparities in the quality of care and services for affected people. Despite the relatively low incidence, tSCIs impose immense burdens on healthcare systems; hospital costs are four times higher than for cardiovascular disease^8^ and 2.8 times higher than for cancer.^9^ This higher cost is driven by long acute (mean 37 days^10^) and rehabilitation (mean 55 days^11^) care stays; in fact, tertiary rehabilitation care costs three times more than inpatient acute care.^12^ Community care is the most challenging stage because people require life-long funding for devices and home adaptations or to access specialized housing, personal support workers, 24/7 nursing supports, and specialized services such as physiotherapy. Traumatic SCI care requires that individuals navigate across different sectors of the healthcare system and that they receive evidence-based care that is informed by high-quality clinical practice guidelines.

Clinical practice guidelines are common resources used to improve practice. The Canadian SCI Practice (Can-SCIP) Guideline^13^ is a good example: it is a living guideline with frequent updates regarding emerging tSCI care evidence designed to facilitate improvements in health outcomes and to assist evidence-based decision-making by healthcare professionals. However, translating guideline recommendations into practice while fitting local models of care is a complex task that can take years,^14^ reducing the positive impacts to persons living with a new or existing tSCI, leading to care inequities, and making healthcare systems less effective and efficient.

Care pathways, in contrast, refer to “structured multidisciplinary care plans which detail essential steps in the care of patients with a specific clinical conditions.”^15^ Steps in this context refer to any timepoint where needs emerge—initial injury, surgery and rehabilitation, or managing cardiovascular issues resulting from reduced mobility post-SCI. Quantifying the gap between ideal and current care, independent of jurisdiction, is difficult due to non-standardized care pathways and inconsistent evaluation strategies used by healthcare systems. Pathways require translation of evidence and existing practice guidelines into specific care maps that provide representations of the step-by-step elements of care and can facilitate the identification of standardized quality indicators. In other words, a well-designed care pathway serves as a “practice guideline” for healthcare system planners and funders and may inform people living with tSCI about what, where, and when care should be received. Pathways were shown to promote shorter lengths of stay, lower cost, and reduce in-hospital complications,^16^ demonstrating their effectiveness in promoting timely, effective, and cost-efficient care and services. Currently, there are no care pathways for tSCI in Canada that cover the entire care continuum.

The objective of this article is to describe the development of an integrated care pathway for tSCI that comprehensively covers the time of injury to lifelong care in the community, utilizing existing evidence sources, clinical consensus, and the experiences of persons living with tSCI and their families. There may be some differences in the pre-acute stages of nontraumatic injuries where spinal damage progression is followed over longer periods because these injuries tend not to be sudden in nature; however, rehabilitation approaches and community needs are quite similar independent of injury etiology. Thus, nontraumatic injuries can also benefit from the tSCI Care Pathway. The tSCI Care Pathway was developed under the umbrella of the Neurotrauma Care Pathways, an initiative funded by the Ontario Ministry of Health and Long-Term Care to develop ideal care pathways for brain and spinal injuries and associated quality indicators.

## Methods

### Key partner engagement

Eighty Ontarian key partners who were invited to co-design the pathways included people with lived experience and their families, clinicians, healthcare administrators, community service providers, researchers, third-party providers (extended healthcare benefits, work insurance, and car collision insurance), injury associations, researchers, public funders, and policymakers. All had experience and expertise related to the tSCI healthcare system in Ontario.

### Project organization and guiding principles

The tSCI Care Pathway development was guided by a Steering Committee; four Working Groups developed the tSCI Care Pathway, and six region-specific Working Groups discussed and identified priorities based on the needs of Ontario, the most populous province in Canada. This work used a quality improvement lens aimed at implementing sustainable best practices at a systemic level. While we did not use any formal qualitative methods of thematic analysis, we followed a similar process as suggested by the Integrated Knowledge Translation (IKT) Guiding Principles^17^: creation of principles, consensus meetings, feedback, and outcome consolidation. In our context, we implemented summits and working meetings (i.e., Steering Committee or Working Groups); took notes; reviewed existing information, literature, or data regarding a given topic; identified common themes that related to the current goals (e.g., meeting focused on creating the guiding principles, meetings focused on identifying gaps in care, meetings focusing on identifying implementation priorities); derived consensus during meetings and/or through surveys; sought further feedback from involved key partners; and summarized the information in written documents (i.e., outcomes). The engagement of persons with lived experience and their families also followed the IKT Guiding Principles: development and maintenance of partnerships based on trust, respect, dignity, and transparency; shared decision-making processes; open and honest communication; valuing all knowledge and expertise; flexible and receptive tailoring of research and development approaches; meaningful mutual benefits; ethical considerations; and respect regarding practical and financial constraints of all partners.

The Steering Committee determined that the tSCI Care Pathway should:

1. Focus on key elements of care or “building blocks” (“the what”; e.g., access to primary care), not specific recommendations for clinicians (“the how”; e.g., hours of therapy).
2. Be person-centred and inclusive of all people living with a tSCI and their families.
3. Be adaptable to varied models of care and address known care gaps (e.g., coordination, transitions, or re-access).
4. Engage key partners who either plan, deliver, or receive care to ensure sustainability and implementability.

### Processes to develop the tSCI Care Pathway

#### Step 1: Identifying existing guidelines, pathways, and models

Systematic searches were executed on PubMed using combinations of terms related to “clinical practice guidelines,” “care pathways,” “models of care,” OR “care maps” AND “spinal cord injury” (traumatic only), from 2011 to 2023. A grey literature search was performed to identify documents not published through traditional peer-review mechanisms. Non-English language references were excluded. We did not perform critical evaluation because it was outside the scope of this project.

#### Step 2: Creating the tSCI Care Pathway

1. *Virtual Summit:* Key partners attended a virtual summit to review the goals and processes for developing the tSCI Care Pathway, summarize the available literature, discuss care gaps, and create Working Groups.
2. *Working Groups* addressed acute care, rehabilitation care, community care, and equity considerations. The groups met virtually to develop building blocks of care. Each Working Group had at least two people with lived experience and representatives from all key partners. The equity considerations group decisions will be briefly mentioned here and are the subject of a separate manuscript.
3. *Drafting the pathway diagram*: All Working Groups identified building blocks for their care stage. The building blocks were linked to existing evidence-based clinical practice guidelines. The gaps between ideal and current practices and available recommendations were discussed with a focus on having them addressed by the chosen building blocks. The equity considerations group reviewed and discussed information related to social determinants of health, marginalized and underserved populations, and care planning and provision and identified potential mitigation strategies for existing barriers.
4. *Validation process*: Further feedback was obtained from persons with lived experience engaged via the Spinal Cord Injury Ontario (SCIO), marginalized and underserved groups, lawyers via the Ontario Trial Lawyers Association, and insurance/extended health care providers via the Insurance Bureau of Canada. Directed questions focused on experiences and whether the drafted pathway addressed the perceived care gaps, pathway advantages and disadvantages, unintended consequences, and potential mitigation strategies for implementation challenges.

#### Step 3: Regional consultations and implementation prioritization

Six additional Working Groups (one for each of the six Ontario Health Regions) addressed regional variabilities in care, services, and needs. Their goals were to identify region-specific challenges to pathway implementation, prioritize these challenges, and identify actionable solutions to drive future implementation. These groups discussed administrative data, perceived importance, regional priorities, and the existence of current services in order to prioritize each challenge/gap. Actionable solutions included highlighting who had to be involved, infrastructure needed, source of funding, advantages and disadvantages of the proposed action, and how to evaluate change. The regional Working Groups had a similar composition to the provincial Working Groups. Twenty-four virtual meetings with 72 Working Group members were held from November 2022 to March 2023, as determined by the work required.

#### Step 4: Development of quality indicators for the tSCI Care Pathway

Using a similar Working Group composition as described previously, members reviewed quality indicators from published sources, developed indicators where none previously existed, and created a core set of quality indicators mapped onto the pathway. The outcomes of this process are briefly described here and will be detailed in a separate article.

## Results

### Step 1 Output: Synthesis of existing guidelines, pathways, and models

Although 107 tSCI-specific clinical practice guidelines (*n* = 67), pathways (*n* = 15), and models of care (*n* = 25) were published from 2011 to 2023, most of them are outdated, reported a single focus (i.e., only a single element of care, such as pressure injury prevention for acute care nurses), and/or did not include equity considerations. Of those, four focused on pre-acute care only, 18 on acute, five on rehabilitation, 18 on community, four on pre-acute and acute, seven on acute and rehabilitation, 37 on rehabilitation and community, and 16 across all stages. The most up-to-date, comprehensive (i.e., holistic view from pre-acute to community care), and relevant documents that supported the tSCI Care Pathway were the Can-SCIP Guideline^13^ (pre-acute to community care clinical practice guideline recommendations), Paralyzed Veterans of America SCI Guidelines (United States; https://pva.org/research-resources/publications/clinical-practice-guidelines/; mostly rehabilitation care guidelines), Spinal Cord Injury Research Evidence (SCIRE; https://scireproject.com/; comprehensive evidence summaries, but not pathway recommendations), and the head and major trauma (i.e., SCI) guidelines from the National Institute for Health and Care Excellence (NICE) Pathways of Care (United Kingdom; detailed care maps for acute and rehabilitation care).^18^ The International Perspectives on Spinal Cord Injury^19^ from the World Health Organization was used as guidance for equity considerations. See eAppendix 1 for the full list of documents reviewed.

### Step 2 Output: tSCI Care Pathway

**Figure 1.**
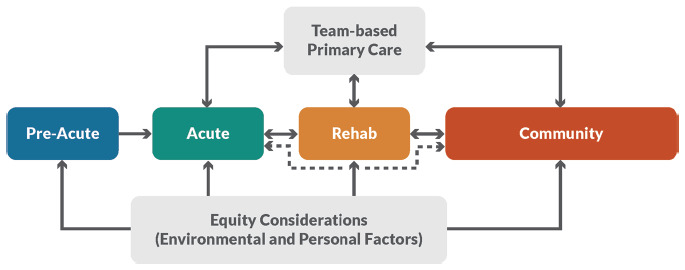
Overarching care pathway.

**Figure 2.**
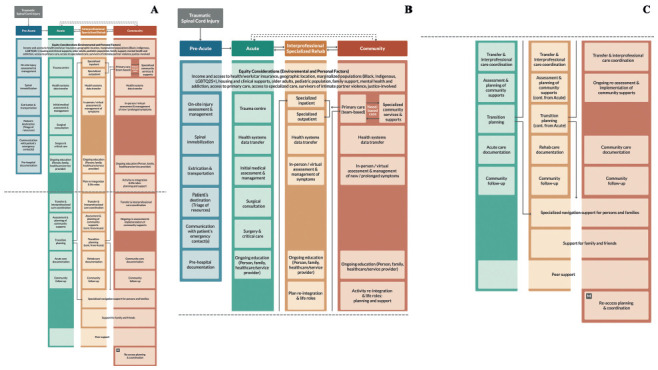
Traumatic spinal cord injury–specific (tSCI) Care Pathway. (**A**) Entire diagram. (**B**) The cropped top part of the diagram. (**C**) The cropped bottom part of the diagram. An interactive version of the tSCI Care Pathway with linkages to clinical practice guidelines and resources can be found at www.neurotraumapathways.ca.

Two formats of the pathway were produced, including an overarching framework that applied to all types of neurotrauma (i.e., concussion, moderate-severe brain injury, and tSCI) (**Figure 1**) and a detailed tSCI Pathway (**Figure 2**).

Key features of the overarching pathway are that each person with a tSCI should have access to specialized care from pre-acute to community care. Even though the pathway appears linear (i.e., Pre-Acute being the entry point and moving straight through Rehabilitation to Community as the exit or final stage), the figure emphasizes the need for people to re-access care and the bidirectional nature of some transitions, for example, between specialized rehabilitation to community depending on personal recovery journey (e.g., secondary complications, ageing, new injury, etc.). Beyond care stages, there are two integral elements: (1) team-based primary care (i.e., interdisciplinary teams) to support the complex needs of people living with tSCI and help them navigate across care stages, especially in the community stage, and (2) equity considerations. The latter refers to personal and social determinants of health such as income, sex, gender, cultural background, being part of a marginalized or underserved group (e.g., Indigenous, Black, LGBTQ2S+), age, geographic location, and familial or housing status and environmental factors such as being part of an incarcerated population, access to insurance/extended healthcare benefits, and concurrent mental illness or addictions.

The tSCI Care Pathway (**Figure 2**) indicates the building blocks selected by the Working Groups. It is important to note that some blocks or elements are relevant to one care stage, and others cut across and are relevant to multiple stages. Building blocks within a single stage should be delivered by teams in that stage and will not carry over to later stages. Building blocks that cut across care stages require continuity of some or all care providers/mechanisms.

Key Acute Care building blocks are timely and appropriate transportation of people from injury site to the most appropriate trauma centre, appropriate initial assessment and management, timely surgical consultations, and interdisciplinary and coordinated transition planning. The Rehabilitation group focused on access to specialized rehabilitation with appropriate assessment and planning of community needs and access to outpatient care. The Community groups highlighted built-in follow-up processes to ensure appropriateness, access and quality of community services, and mechanisms to re-access acute and rehabilitation care as needs change over time.

All Working Groups emphasized the need for ongoing education for providers and patients and the need for collecting data across stages to understand health outcomes and care plans and to reduce duplication of efforts. Persons with lived experience emphasized (1) the value of introducing peer support early in acute care, but with multiple access points post discharge, (2) the value of focusing specialized rehabilitation on gaining skills and expertise related to self-management and activities of daily living, (3) the need for rehabilitation strategies promoting maintenance and continuation of gains post specialized rehabilitation, and (4) the importance of having supports (e.g. vocational, educational, hobbies, volunteering) to better facilitate community and life role participation. Families emphasized the personal burden of caring for their loved ones and the current lack of care and supports; building blocks were added to the model focusing on families and carers’ needs. Equity considerations were identified and incorporated in all care stages.

The Neurotrauma Care Pathways Project Team integrated and linked the building blocks with the content of clinical practice guidelines. Other tools and resources were identified for building blocks that lacked associated clinical practice guidelines, for example, the lack of equity considerations in most clinical practice guidelines and guidance on how to support families appropriately.

### Step 3 Output: Regionalized pathways and implementation priorities

**Figure 3.**
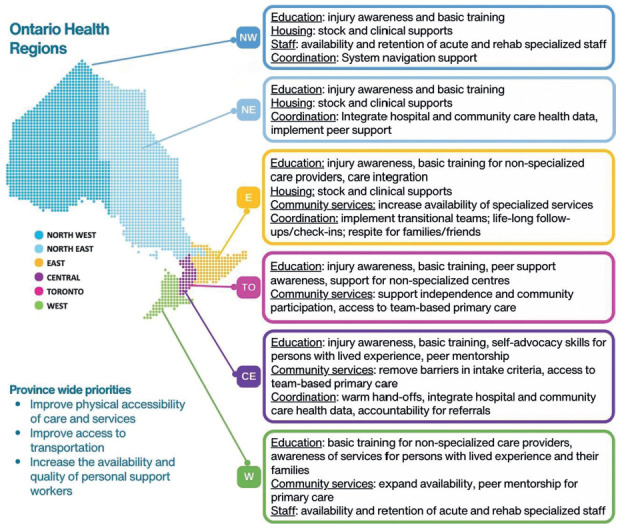
Summary of regional priorities across the six Ontario Health Regions. NW: north west, NE: north east, E: east, TO: Toronto, CE: central, and W: west.

Regional Working Groups identified 12 feasible regional priority gaps (see **Figure 3**). All regions prioritized ongoing education, access to specialized navigation support, and expansion of the availability of community services. These complement the known provincial priorities that include improving accessibility of the built environment, availability of adapted transportation in public and private sectors, and availability and tSCI-specific skills of personal support workers. It was recognized that it would take time to fully implement the pathway and eliminate gaps; as a start, groups developed regional action plans to mitigate/eliminate current gaps in priority areas (For details, see eAppendix 2.)

### Step 4 Output: Development of quality indicators

Seventy-one quality indicators were developed that map onto all stages of the care pathway to measure system performance. Indicators were created based on indicators from existing literature and published quality indicators used in clinical practice and knowledge gaps in quality of care. Data cannot be currently collected for all the indicators, so a core set of 24 critical and feasible indicators were prioritized for immediate implementation. Details about the quality indicators will be published separately and will be publicly available on our website (www.neurotraumapathways.ca).

## Discussion

This is the first comprehensive care pathway for tSCI in Canada and one of the most detailed worldwide. The tSCI Care Pathway fills a gap in guidance by providing clarity on what care and services are needed from time of injury to life in the community and mitigates the causes of inequitable care provision and compromised health outcomes. It is grounded in the best practices from current clinical practice guidelines, clinical consensus, and the opinions of persons with lived experience, while integrating equity considerations across all stages. Thus, the tSCI Care Pathway focuses on facilitating “the right care, at the right time, by the right team and in the right place.”^20^ Although the tSCI Care Pathway was produced in one province, it has been used as a blueprint for pathway work in British Columbia and is informing the development of a national SCI strategy.

There are some important differences and similarities between our work and those of other countries. The closest similarities exist in the acute and rehabilitation care building blocks in the pathways adopted in Queensland (Australia),^21^ Ireland,^22^ and United Kingdom.^18^ Pathways are different from “models of care” in that pathways provide navigation guidance that links the core care elements, whereas a model of care typically pertains to one or two care sectors (e.g. Acute and Rehabilitation) or to the jurisdictional view of care provision (i.e., from a governmental perspective) and do not always address the reality that people with tSCI face with transitions across time, providers, organizations, and geographical locations.^23^

Other key differences between this pathway and other pathways and models of care include the following:

1. The tSCI Pathway is adaptable because it focuses on fundamental needs and requirements of the overarching healthcare system and not on influencing the ground-level practice of clinicians.
2. We have conscientiously linked the blocks to the underlying clinical practice guidelines and considered the social determinants of health/equity issues.
3. The tSCI Pathway can adapt to the needs of marginalized and underserved populations, addressing different entry points, needs, interdisciplinary care coordination, and re-access once people return to the community.
4. We have created quality indicators to evaluate fundamental systemic care elements across care stages, including equity.

In contrast, when we look at other similar initiatives, the majority focus on acute care or rehabilitation care only. Most existing pathways and models of care lack equity considerations and do not provide detailed guidance about care and services needed in the community and across the lifespan. The tSCI Care Pathway is a considerable advancement in the approach to provide integrated and equitable care across the lifespan for tSCI.

There are some limitations to this work. We targeted the initial care pathway for traumatic injuries since most clinical practice guidelines focus on this population. Moving forward, we will include nontraumatic injuries, acknowledging that the acute care stage may differ. We used clinical consensus and the experience of persons living with tSCI in our province when identifying building blocks, and it is possible that all elements may not be generalizable to all jurisdictions. Although the tSCI Care Pathway is adaptable, it may need to be further adapted to account for different cultural and socioeconomic settings in different countries where conceptualizations of health, rehabilitation, and community living may be different. The tSCI Care Pathway is an excellent guiding document at a health systems level, but there is a need for initiatives working at a more detailed level; a great example is the SCI Implementation, Evaluation & Quality Care Consortium, which is implementing and evaluating specialized SCI rehabilitation best practices in Ontario. A crucial next step in implementing the regional priorities is to test the core set of quality indicators in evaluating the success of implementation and provide feedback and reporting.

## Conclusion

The tSCI Care Pathway is a guideline to facilitate system transformation targeting high-quality and equitable care. The Pathway is inclusive to all, independent of location of residence, income, or population group; it is adaptable to varied models of care; and it covers elements from time of injury to needs across the lifespan. Policymakers and administrators can use the tSCI Care Pathways and associated quality indicators to plan healthcare priorities, design and evaluate healthcare systems, and allocate funds. Clinicians and community care and service providers can take advantage of the linkage with clinical practice guidelines. Persons with lived experience and their families benefit from a better understanding of the injury, next steps, expectations, needed education, and available supports across the lifespan, with the overall goal of improving health outcomes, self-management, and community participation for all.

## Supplementary Material




