# Supplementary material for: What Is the Pathway to the Best Model of Care for Traumatic Spinal Cord Injury? Evidence-Based Guidance
Source: Top Spinal Cord Inj Rehabil. 2023 Nov 17;29(Suppl):103–11. doi: 10.46292/sci23-00059S (PMC10759857; doi:10.46292/sci23-00059S)
Supplement: Supplementary file 1 [file i1945-5763-29-suppl-103_s01.pdf]

eAPPENDIX 1

eTable 1. Summary of the literature search for clinical practice guidelines (CPG), pathways, and models of care (MoC) related to tSCI

Main author (region)	Publication year	Title	Care stage	Type
Primary sources				
Patsakos (Canada)[1]	2021	Evaluation of the quality of published SCI clinical practice guidelines using the AGREE II instrument: Results from Can-SCIP expert panel	All stages	CPG
NICE (UK)[2]	2016	Spinal Injury: Assessment and Initial Management	Acute & Rehabilitation	Pathway
Spinal Cord Injury Model Systems[3]	2021	Resources offered by the MSKTC to support individuals living with spinal cord injury	All stages	CPG
Secondary sources				
Ahn (Canada)[4]	2011	Pre-hospital care management of a potential spinal cord injured patient: A systematic review of the literature and evidence-based guidelines	Pre-Acute	CPG
Albert (France)[5]	2012	Physical and rehabilitation medicine (PRM) care pathways: "spinal cord injury"	Rehabilitation	Pathway
Albert (France)[5]	2012	Physical and rehabilitation medicine (PRM) care pathways: "spinal cord injury"	All stages	Pathway
Allan (Canada)[6]	2018	Simplified guideline for prescribing medical cannabinoids in primary care	Community	CPG
Barclay (International)[7]	2020	A comparative examination of models of service delivery intended to support community integration in the immediate period following inpatient rehabilitation for spinal cord injury	Community	MoC
Barclay (Australia)[8]	2019	A scoping review of peer-led interventions following spinal cord injury	Rehabilitation & Community	MoC
Bertschy (Germany)[9]	2020	Guideline for the management of pre-, intra-, and postpartum care of women with a spinal cord injury	Community	CPG
Bickenbach (International)[10]	2013	International Perspectives on Spinal Cord Injury. Geneva: World Health Organization	All stages	Pathway

(continues)

This supplementary digital material accompanies
Top Spinal Cord Inj Rehabil 2023;29(suppl):103-111
© 2023 American Spinal Injury Association
www.asia-spinalinjury.org
doi: 10.46292/sci23-00059S

eTable 1. Summary of the literature search for clinical practice guidelines (CPG), pathways, and models of care (MoC) related to tSCI *(cont.)*

Main author (region)	Publication year	Title	Care stage	Type
Bombardier (USA)[11]	2021	Management of Mental Health Disorders, Substance Use Disorders, and Suicide in Adults with Spinal Cord Injury: Clinical Practice Guideline for Healthcare Providers	Community	CPG
Botchway (Australia)[12]	2022	Rehabilitation models of care for children and youth living with traumatic brain and/or spinal cord injuries: A focus on family-centred care, psychosocial wellbeing, and transitions	Rehabilitation & Community	MoC
Caremel (France)[13]	2013	Expert opinion on surgical care pathway management of neurologic patients from Neuro-Urology Committee of the French National Association of Urology (AFU)	Acute	Pathway
Chhabra (International)[14]	2019	Stem cell/cellular interventions in human spinal cord injury: Is it time to move from guidelines to regulations and legislations? Literature review and Spinal Cord Society position statement	Acute	CPG
Consortium for Spinal Cord Medicine (USA)[15]	2016	Prevention of Venous Thromboembolism in Individuals with Spinal Cord Injury: Clinical Practice Guidelines for Health Care Providers, 3rd ed.	Acute & Rehabilitation	CPG
Consortium for Spinal Cord Medicine (USA)[16]	2010	Sexuality and reproductive health in adults with spinal cord injury: A clinical practice guideline for health-care professionals	Rehabilitation & Community	CPG
Cotterill (International)[17]	2018	Neurogenic bowel dysfunction: Clinical management recommendations of the Neurologic Incontinence Committee of the Fifth International Consultation on Incontinence 2013	Rehabilitation & Community	CPG
Craig (Australia)[18]	2013	Guide for Health Professionals for Psychosocial Care of Adults with Spinal Cord Injury	Rehabilitation & Community	CPG
Deer (International)[19]	2017	The Neurostimulation Appropriateness Consensus Committee (NACC) Safety Guidelines for the Reduction of Severe Neurological Injury	Acute & Rehabilitation	CPG
DeJong (USA)[20]	2011	Postrehabilitative Health Care for Individuals with SCI: Extending Health Care into the Community	Community	MoC

(continues)

eTable 1. Summary of the literature search for clinical practice guidelines (CPG), pathways, and models of care (MoC) related to tSCI *(cont.)*

Main author (region)	Publication year	Title	Care stage	Type
Department of Veterans Affairs (USA)[21]	2019	Spinal Cord Injuries and Disorders System of Care	All stages	MoC
Dhall (USA)[22]	2013	Deep venous thrombosis and thromboembolism in patients with cervical spinal cord injuries	Rehabilitation & Community	CPG
EnableNSW (Australia)[23]	2011	Development of Clinical Guidelines for the Prescription of a Seated Wheelchair or Mobility Scooter for People with TBI or SCI	Rehabilitation & Community	CPG
Farkas (USA)[24]	2021	Energy expenditure and nutrient intake after spinal cord injury: A comprehensive review and practical recommendations	Rehabilitation & Community	CPG
Fehlings (International)[25]	2017	A Clinical Practice Guideline for the Management of Patients with Acute Spinal Cord Injury: Recommendations on the Role of Baseline Magnetic Resonance Imaging in Clinical Decision Making and Outcome Prediction	Acute	CPG
Fehlings (International)[26]	2017	A Clinical Practice Guideline for the Management of Patients with Acute Spinal Cord Injury: Recommendations on the Type and Timing of Anticoagulant Thromboprophylaxis	Acute	CPG
Fehlings (International)[27]	2017	A Clinical Practice Guideline for the Management of Patients with Acute Spinal Cord Injury: Recommendations on the Type and Timing of Rehabilitation	Acute	CPG
Fehlings (International)[28]	2017	A Clinical Practice Guideline for the Management of Patients with Acute Spinal Cord Injury and Central Cord Syndrome: Recommendations on the Timing (≤24 Hours Versus >24 Hours) of Decompressive Surgery	Acute	CPG
Fehlings (International)[29]	2017	A Clinical Practice Guideline for the Management of Patients with Acute Spinal Cord Injury: Recommendations on the Use of Methylprednisolone Sodium Succinate	Acute	CPG

(continues)

eTable 1. Summary of the literature search for clinical practice guidelines (CPG), pathways, and models of care (MoC) related to tSCI *(cont.)*

Main author (region)	Publication year	Title	Care stage	Type
Franz (Germany)[30]	2019	Management of pain in individuals with spinal cord injury: Guideline of the German-Speaking Medical Society for Spinal Cord Injury	Rehabilitation & Community	CPG
Garber (USA)[31]	2014	Pressure Ulcer Prevention and Treatment Following Spinal Cord Injury: A Clinical Practice Guideline for Health-Care Professionals, 2nd ed.	Acute & Rehabilitation	CPG
Gelb (USA)[32]	2013	Initial closed reduction of cervical spinal fracture-dislocation injuries	Acute	CPG
Gelis (France)[33]	2018	French guidelines from PERSE, SoFCPRE, and SOFMER for the medical and surgical management of pressure ulcers in persons with spinal cord injury	Acute & Rehabilitation	CPG
Groen (Europe)[34]	2016	Summary of European Association of Urology (EAU) Guidelines on Neuro-Urology	All stages	CPG
Guidelines and Audit Implementation Network (UK)[35]	2014	Guidelines for the Rehabilitation of Patients with Metastatic Spinal Cord Compression (MSCC): Assessment and Care Provision by Occupational Therapists and Physiotherapists in the Acute Sector	Acute & Rehabilitation	CPG
Guy (Canada)[36]	2016	The CanPain SCI Clinical Practice Guideline for Rehabilitation Management of Neuropathic Pain after Spinal Cord: Recommendations for model systems of care	Rehabilitation & Community	CPG
Health Service Executive (Ireland)[37]	2018	Integrated Care Pathway for the Management of Spinal Cord Injury	All stages	Pathway
Hentzen (France)[38]	2022	Prioritization of risk situations in neuro-urology: Guidelines from Association Française d'Urologie (AFU), Association Francophone Internationale des Groupes d'Animation de la Paraplégie (A.F.I.G.A.P.), Groupe de Neuro-urologie de Langue Française (GENULF), Société Française de Médecine Physique et de Réadaptation (SOFMER) and Société Interdisciplinaire Francophone d'UroDynamique et de Pelvi-Périnéologie (SIFUD-PP)	Rehabilitation & Community	CPG

(continues)

eTable 1. Summary of the literature search for clinical practice guidelines (CPG), pathways, and models of care (MoC) related to tSCI *(cont.)*

**Main author (region)**	**Publication year**	**Title**	**Care stage**	**Type**
Ho (Canada)[39]	2021	Models of Care Delivery from Rehabilitation to Community for Spinal Cord Injury: A Scoping Review	Rehabilitation & Community	MoC
Hornby (USA)[40]	2020	Clinical Practice Guideline to Improve Locomotor Function Following Chronic Stroke, Incomplete Spinal Cord Injury, and Brain Injury	Rehabilitation & Community	CPG
Houghton (Canada)[41]	2019	Canadian Best Practice Guidelines for the Prevention and Management of Pressure Ulcers in People with Spinal Cord Injury	Rehabilitation & Community	CPG
Johns (USA)[42]	2021	Management of Neurogenic Bowel Dysfunction in Adults after Spinal Cord Injury: Clinical Practice Guideline for Health Care Providers	Rehabilitation & Community	CPG
Jones (USA)[43]	2019	Peer mentoring reduces unplanned readmissions and improves self-efficacy following inpatient rehabilitation for individuals with spinal cord injury	Community	MoC
Kavanagh (Canada)[44]	2019	Canadian Urological Association guideline: Diagnosis, management, and surveillance of neurogenic lower urinary tract dysfunction	Rehabilitation & Community	CPG
Kleeman (Australia)[45]	2018	Mapping the continuum of care to surgery following traumatic spinal cord injury	Pre-Acute & Acute	Pathway
Kornhall (Norway)[46]	2017	The Norwegian guidelines for the prehospital management of adult trauma patients with potential spinal injury	Pre-Acute	CPG
Krassioukov (Canada)[47]	2012	International standards to document remaining autonomic function after spinal cord injury	Rehabilitation & Community	CPG
Krassioukov (Canada)[48]	2021	Evaluation and Management of Autonomic Dysreflexia and Other Autonomic Dysfunctions: Preventing the Highs and Lows: Management of Blood Pressure, Sweating, and Temperature Dysfunction	Rehabilitation & Community	CPG
Lala (Canada)[49]	2016	Developing a Model of Care for Healing Pressure Ulcers with Electrical Stimulation Therapy for Persons With Spinal Cord Injury	Rehabilitation & Community	MoC

(continues)

eTable 1. Summary of the literature search for clinical practice guidelines (CPG), pathways, and models of care (MoC) related to tSCI *(cont.)*

Main author (region)	Publication year	Title	Care stage	Type
Lamontagne (Canada)[50]	2019	Implementation Evaluation of an Online Peer-Mentor Training Program for Individuals with Spinal Cord Injury	Community	MoC
Lee (Canada)[51]	2014	Evaluation of a primary care-based mobility clinic: improving health care for individuals with mobility impairments in Ontario, Canada.	Community	MoC
Li (China)[52]	2021	Effects of Online Home Nursing Care Model Application on Patients with Traumatic Spinal Cord Injury	Community	MoC
Loh (Canada)[53]	2022	The CanPain SCI clinical practice guidelines for rehabilitation management of neuropathic pain after spinal cord injury: 2021 update	Rehabilitation	CPG
Lukersmith (Australia)[54]	2013	Development of clinical guidelines for the prescription of a seated wheelchair or mobility scooter for people with traumatic brain injury or spinal cord injury	Rehabilitation & Community	CPG
Madaris (USA)[55]	2016	SCI Hospital in Home Program: Bringing Hospital Care Home for Veterans with Spinal Cord Injury	Community	MoC
Martin Ginis (International)[56]	2018	Evidence-based scientific exercise guidelines for adults with spinal cord injury: an update and a new guideline	Rehabilitation & Community	CPG
Maschmann (Denmark)[57]	2019	New clinical guidelines on the spinal stabilisation of adult trauma patients - consensus and evidence based	Pre-Acute	CPG
McColl (Canada)[58]	2012	Primary care of people with spinal cord injury: Scoping review	Community	MoC
McKim (Canada)[59]	2011	Home mechanical ventilation: a Canadian Thoracic Society clinical practice guideline	Community	CPG
Mehta (Canada)[60]	2016	The CanPain SCI Clinical Practice Guidelines for Rehabilitation Management of Neuropathic Pain after Spinal Cord: Screening and diagnosis recommendations	Rehabilitation & Community	CPG
Middleton (Australia)[61]	2014	Right care, right time, right place: Improving outcomes for people with spinal cord injury through early access to intervention and improved access to specialised care: study protocol	All stages	Pathway

(continues)

eTable 1. Summary of the literature search for clinical practice guidelines (CPG), pathways, and models of care (MoC) related to tSCI *(cont.)*

Main author (region)	Publication year	Title	Care stage	Type
Milligan (Canada)[62]	2016	Enhancing primary care for persons with spinal cord injury: More than improving physical accessibility	Community	MoC
Ministry of Health (New Zealand)[63]	2014	New Zealand Spinal Cord Impairment Action Plan 2014–2019	All stages	MoC
Morse (USA)[64]	2019	Bone Mineral Density Testing in Spinal Cord Injury: 2019 ISCD Official Position	Rehabilitation & Community	CPG
Multidisciplinary Association of Spinal Cord Injury Professionals (UK & Ireland)[65]	2013	Clinical Guideline for Standing in Adults Following Spinal Cord Injury	Rehabilitation & Community	CPG
Nash (USA)[66]	2018	Identification and Management of Cardiometabolic Risk after Spinal Cord Injury: Clinical Practice Guideline for Health Care Providers	Rehabilitation & Community	CPG
Nash (USA)[67]	2019	Identification and Management of Cardiometabolic Risk after Spinal Cord Injury	Rehabilitation & Community	CPG
National Health Services Spinal Cord Injury Services (UK)[68]	2019	Service Specification for the Provision of Spinal Cord Injuries Services	All stages	MoC
Neurosurgery Educator and Outreach Network (Canada)[69]	2016	Provincial Guidelines for Spinal Cord Assessment	Acute	CPG
New South Wales Agency for Clinical Innovation (Wales)[70]	2017	Report on Spinal Cord Injury Model of Care	All stages	MoC
NICE (UK)[71]	2020	Neuropathic pain in adults: Pharmacological management in non-specialist settings	Community	CPG

(continues)

eTable 1. Summary of the literature search for clinical practice guidelines (CPG), pathways, and models of care (MoC) related to tSCI *(cont.)*

Main author (region)	Publication year	Title	Care stage	Type
NICE (UK)[72]	2012	Urinary Incontinence in Neurological Disease: Management of Lower Urinary Tract Dysfunction in Neurological Disease	Rehabilitation & Community	CPG
Nicolle (USA)[73]	2019	Clinical Practice Guideline for the Management of Asymptomatic Bacteriuria: 2019 Update by the Infectious Diseases Society of America	Rehabilitation & Community	CPG
Noonan (Canada)[74]	2012	The Application of Operations Research Methodologies to the Delivery of Care Model for Traumatic Spinal Cord Injury: The Access to Care and Timing Project	All stages	MoC
Norton (Canada)[75]	2017	Best practice recommendations for the prevention and management of pressure injuries	Rehabilitation & Community	CPG
Nuwer (USA)[76]	2012	Evidence-based guideline update: Intraoperative spinal monitoring with somatosensory and transcranial electrical motor evoked potentials	Acute	CPG
O'Dell (UK)[77]	2018	Role of peer support for people with a spinal cord injury	Rehabilitation & Community	MoC
Osipowicz (USA)[78]	2023	Emergency Department and PICU Clinical Pathway for Evaluation/Treatment of Children with Suspected Traumatic Spinal Cord Injury	Pre-Acute & Acute	Pathway
Osman (UK)[79]	2017	The evolution of national care pathways in spinal cord injury management	All stages	Pathway
Osmonov (Europe)[80]	2020	Clinical Recommendations from the European Society for Sexual Medicine Exploring Partner Expectations, Satisfaction in Male and Phalloplasty Cohorts, the Impact of Penile Length, Girth and Implant Type, Reservoir Placement, and the Influence of Comorbidities and Social Circumstances	Rehabilitation & Community	CPG
Picinich (USA)[81]	2019	Activation to Arrival: Transition and Handoff from Emergency Medical Services to Emergency Departments	Pre-Acute & Acute	Pathway
Pilusa (South Africa)[82]	2022	Services and interventions needed to prevent secondary health conditions throughout the life span of people with spinal cord injury, South Africa	Community	MoC

(continues)

eTable 1. Summary of the literature search for clinical practice guidelines (CPG), pathways, and models of care (MoC) related to tSCI *(cont.)*

Main author (region)	Publication year	Title	Care stage	Type
Prange-Lasonder (Europe)[83]	2019	European evidence-based recommendations for clinical assessment of upper limb in neurorehabilitation (CAULIN): Data synthesis from systematic reviews, clinical practice guidelines and expert consensus	Rehabilitation	CPG
Princess Alexandra Hospital (Australia)[84]	2018	Queensland Spinal Cord Injuries Service Model of Care	All stages	MoC
Quddusi (Canada)[85]	2023	Early surgical intervention for acute spinal cord injury: Time is spine	Acute	CPG
Rapidi (Europe)[86]	2018	Evidence-based position paper on Physical and Rehabilitation Medicine (PRM) professional practice for persons with spinal cord injury. The European PRM position (UEMS PRM Section)	Rehabilitation & Community	CPG
Roquilly (France)[87]	2020	French recommendations for the management of patients with spinal cord injury or at risk of spinal cord injury	Acute & Rehabilitation	CPG
Rudden (Canada)[88]	2018	Pediatric Spinal Cord Injury Clinical Pathway	All stages	Pathway
Ruediger (USA)[89]	2019	Decreasing re-hospitalizations and emergency department visits in persons with recent spinal cord injuries using a specialized medical home	Community	MoC
Russell (USA)[90]	2020	Professional standards of practice for psychologists, social workers, and counselors in SCI rehabilitation	Rehabilitation & Community	CPG
Ryken (USA)[91]	2013	Management of acute combination fractures of the atlas and axis in adults	Acute	CPG
Santos (Canada)[92]	2013	Modeling the patient journey from injury to community reintegration for persons with acute traumatic spinal cord injury in a Canadian centre	All stages	MoC
Schurch (International)[93]	2018	Urodynamics in patients with spinal cord injury: A clinical review and best practice paper by a working group of The International Continence Society Urodynamics Committee	Rehabilitation	CPG

(continues)

eTable 1. Summary of the literature search for clinical practice guidelines (CPG), pathways, and models of care (MoC) related to tSCI *(cont.)*

Main author (region)	Publication year	Title	Care stage	Type
Sethi (USA)[94]	2020	Team Approach: Safety and Value in the Practice of Complex Adult Spinal Surgery	Acute	MoC
Stansby (UK)[95]	2014	Prevention and management of pressure ulcers in primary and secondary care: summary of NICE guidance	Rehabilitation & Community	CPG
Stein (USA)[96]	2017	Emergency Neurological Life Support: Traumatic Spine Injury	Pre-Acute & Acute	CPG
Tremont (USA)[97]	2022	Acute Traumatic Spinal Cord Injury: Implementation of a Multidisciplinary Care Pathway	Acute	Pathway
Tweedy (Australia)[98]	2017	Exercise and sports science Australia (ESSA) position statement on exercise and spinal cord injury	Rehabilitation & Community	CPG
Vaikuntam (Australia)[99]	2019	Identifying Predictors of Higher Acute Care Costs for Patients with Traumatic Spinal Cord Injury and Modeling Acute Care Pathway Redesign: A Record Linkage Study	Acute	Pathway
Vaikuntam (Australia)[100]	2018	Assessing the impact of care pathways on potentially preventable complications and costs for spinal trauma patients: protocol for a data linkage study using cohort study and administrative data	Acute	Pathway
Van de pol (Australia)[101]	2016	The delivery of specialist spinal cord injury services in Queensland and the potential for telehealth	Community	MoC
van der Scheer (International)[102]	2021	Functional electrical stimulation cycling exercise after spinal cord injury: A systematic review of health and fitness-related outcomes	Rehabilitation & Community	CPG
Walia (Canada)[103]	2019	Facilitators and Barriers for Implementing an Internet Clinic for the Treatment of Pressure Injuries	Community	MoC
Walters (USA)[104]	2013	Guidelines for the management of acute cervical spine and spinal cord injuries: 2013 update	Acute	CPG
Yli-Hankala (Scandinavian)[105]	2021	Clinical practice guideline on spinal stabilisation of adult trauma patients: Endorsement by the Scandinavian Society of Anaesthesiology and Intensive Care Medicine	Pre-Acute	CPG

(continues)

eTable 1. Summary of the literature search for clinical practice guidelines (CPG), pathways, and models of care (MoC) related to tSCI *(cont.)*

Main author (region)	Publication year	Title	Care stage	Type
Yue (USA)[106]	2016	A review and update on the guidelines for the acute management of cervical spinal cord injury - Part II	Acute	CPG
Zehr (Canada)[107]	2011	Evidence-based risk assessment and recommendations for physical activity clearance: stroke and spinal cord injury	Rehabilitation & Community	CPG

eTable 2. Summary of literature type of document

Literature type	Total
Clinical Practice Guideline (CPG)	67
Pathway	15
Model of Care (MoC)	27

eTable 3. Summary of literature search outcomes by care stage

Literature care stage	Total
Pre-Acute	4
Acute	18
Rehabilitation	5
Community	18
Pre-Acute & Acute	4
Acute & Rehabilitation	7
Rehabilitation & Community	37
All stages	16

REFERENCES

1. Patsakos EM, Craven BC, Kua A, et al. Evaluation of the quality of published SCI clinical practice guidelines using the AGREE II instrument: Results from Can-SCIP expert panel. *J Spinal Cord Med*. 2021;44(sup1):S69-S78.
2. National Institute for Health and Care Excellence (NICE). *Spinal Injury: Assessment and Initial Management*. 2016.
3. Spinal Cord Injury Model Systems. *Resources Offered by the MSKTC to Support Individuals Living With Spinal Cord Injury*. 7th ed. Arlington, VA: Model Systems Knowledge Translation Center; 2021.
4. Ahn H, Singh J, Nathens A, et al. Pre-hospital care management of a potential spinal cord injured patient: a systematic review of the literature and evidence-based guidelines. *J Neurotrauma*. 2011;28(8):1341-1361.
5. Albert T, Beuret Blanquart F, Le Chapelain L, et al. Physical and rehabilitation medicine (PRM) care pathways: "spinal cord injury." *Ann Phys Rehabil Med*. 2012;55(6):440-450.
6. Allan GM, Ramji J, Perry D, et al. Simplified guideline for prescribing medical cannabinoids in primary care. *Can Fam Physician*. 2018;64(2):111-120.

7. Barclay L, Lalor A, Migliorini C, Robins L. A comparative examination of models of service delivery intended to support community integration in the immediate period following inpatient rehabilitation for spinal cord injury. *Spinal Cord*. 2020;58(5):528-536.
8. Barclay L, Hilton GM. A scoping review of peer-led interventions following spinal cord injury. *Spinal Cord*. 2019;57(8):626-635.
9. Bertschy S, Schmidt M, Fiebag K, Lange U, Kues S, Kurze I. Guideline for the management of pre-, intra-, and postpartum care of women with a spinal cord injury. *Spinal Cord*. 2020;58(4):449-458.
10. World Health Organization. *International Perspectives on Spinal Cord Injury*. Geneva: World Health Organization; 2013.
11. Bombardier CH, Azuero CB, Fann JR, Kautz DD, Richards JS, Sabharwal S. Management of mental health disorders, substance use disorders, and suicide in adults with spinal cord injury: Clinical practice guideline for healthcare providers. *Top Spinal Cord Inj Rehabil*. 2021;27(2):152-224.
12. Botchway EN, Knight S, Muscara F, et al. Rehabilitation models of care for children and youth living with traumatic brain and/or spinal cord injuries: A focus on family-centred care, psychosocial wellbeing, and transitions. *Neuropsychol Rehabil*. 2022;32(4):537-559.
13. Caremel R, Phe V, Bart S, et al. [Expert opinion on surgical care pathway management of neurologic patients from Neuro-Urology Committee of the French National Association of Urology (AFU)]. *Prog Urol*. 2013;23(5):309-16.
14. Chhabra HS, Sarda K, Jotwani G, et al. Stem cell/cellular interventions in human spinal cord injury: Is it time to move from guidelines to regulations and legislations? Literature review and Spinal Cord Society position statement. *Eur Spine J*. 2019;28(8):1837-1845.
15. Consortium for Spinal Cord Medicine. Prevention of venous thromboembolism in individuals with spinal cord injury: Clinical practice guidelines for health care providers, 3rd ed.: Consortium for Spinal Cord Medicine. *Top Spinal Cord Inj Rehabil*. 2016;22(3):209-240.
16. Consortium for Spinal Cord Medicine. Sexuality and reproductive health in adults with spinal cord injury: A clinical practice guideline for health-care professionals. *J Spinal Cord Med*. 2010;33(3):281-336.
17. Cotterill N, Madersbacher H, Wyndaele JJ, et al. Neurogenic bowel dysfunction: Clinical management recommendations of the Neurologic Incontinence Committee of the Fifth International Consultation on Incontinence 2013. *Neurourol Urodyn*. 2018;37(1):46-53.
18. Craig A, Perry KN. *Guide for Health Professionals for Psychosocial Care of Adults With Spinal Cord Injury*. 2nd ed. NSW Agency for Clinical Innovation; 2013.
19. Deer TR, Lamer TJ, Pope JE, et al. The Neurostimulation Appropriateness Consensus Committee (NACC) Safety guidelines for the reduction of severe neurological injury. *Neuromodulation*. 2017;20(1):15-30.
20. DeJong G, Hoffman J, Meade M, et al. Postrehabilitative health care for individuals with SCI: Extending health care into the community. *Top Spinal Cord Inj Rehabil*. 2011;17(2):46-58.
21. Veterans Health Administration. *Spinal Cord Injuries and Disorders System of Care*. 2020. www.va.gov/vhapublications/ViewPublication.asp?pub_ID=8523
22. Dhall SS, Hadley MN, Aarabi B, et al. Deep venous thrombosis and thromboembolism in patients with cervical spinal cord injuries. *Neurosurgery*. 2013;72(suppl 2):244-254.
23. Lukersmith S, Radbron L, Hopman K. Development of clinical guidelines for the prescription of a seated wheelchair or mobility scooter for people with traumatic brain injury or spinal cord injury. *Aust Occup Ther J*. 2013; 60(6):378-86.
24. Farkas GJ, Sneij A, McMillan DW, Tiozzo E, Nash MS, Gater DR. Energy expenditure and nutrient intake after spinal cord injury: A comprehensive review and practical recommendations. *Br J Nutr*. 2021:1-25.
25. Fehlings MG, Martin AR, Tetreault LA, et al. A Clinical practice guideline for the management of patients with acute spinal cord injury: Recommendations on the role of baseline magnetic resonance imaging in clinical decision making and outcome prediction. *Global Spine J*. 2017;7(3 suppl):221S-230S.
26. Fehlings MG, Tetreault LA, Aarabi B, et al. A clinical practice guideline for the management of patients with acute spinal cord injury: Recommendations on the type and timing of anticoagulant thromboprophylaxis. *Global Spine J*. 2017;7(3 suppl):212S-220S.
27. Fehlings MG, Tetreault LA, Aarabi B, et al. A clinical practice guideline for the management of patients with acute spinal cord injury: Recommendations on the type and timing of rehabilitation. *Global Spine J*. 2017;7(3 suppl):231S-238S.
28. Fehlings MG, Tetreault LA, Wilson JR, et al. A clinical practice guideline for the management of patients with acute spinal cord injury and central cord syndrome: Recommendations on the timing (≤24 hours versus >24 hours) of decompressive surgery. *Global Spine J*. 2017;7(3 suppl):195S-202S.
29. Fehlings MG, Wilson JR, Tetreault LA, et al. A clinical practice guideline for the management of patients with acute spinal cord injury: Recommendations on the use of methylprednisolone sodium succinate. *Global Spine J*. 2017;7(3 suppl):203S-211S.
30. Franz S, Schulz B, Wang H, et al. Management of pain in individuals with spinal cord injury: Guideline of the German-Speaking Medical Society for Spinal Cord Injury. *Ger Med Sci*. 2019;17:Doc05.
31. Garber SL. *Pressure Ulcer Prevention and Treatment Following Spinal Cord Injury: A Clinical Practice*

Guideline for Health-Care Professionals. 2nd ed. Consortium for Spinal Cord Medicine; 2014.
32. Gelb DE, Hadley MN, Aarabi B, et al. Initial closed reduction of cervical spinal fracture-dislocation injuries. *Neurosurgery*. 2013;72(suppl 2):73-83.
33. Gelis A, Colin D, Perrouin-Verbe B, et al. French guidelines from PERSE, SoFCPRE and SOFMER for the medical and surgical management of pressure ulcers in persons with spinal cord injury. *Ann Phys Rehabil Med*. 2018;61(5):352-354.
34. Groen J, Pannek J, Castro Diaz D, et al. Summary of European Association of Urology (EAU) Guidelines on Neuro-Urology. *Eur Urol*. 2016;69(2):324-333.
35. Guidelines and Audit Implementation Network. *Guidelines for the Rehabilitation of Patients with Metastatic Spinal Cord Compression (MSCC): Assessment and Care Provision by Occupational Therapists and Physiotherapists in the Acute Sector*. Northern Ireland: Guidelines and Audit Implementation Network; 2014.
36. Guy SD, Mehta S, Harvey D, et al. The CanPain SCI Clinical Practice Guideline for Rehabilitation Management of Neuropathic Pain after Spinal Cord: Recommendations for model systems of care. *Spinal Cord*. 2016;54(Suppl 1):S24-7.
37. Health Service Executive. *Integrated Care Pathway for the Management of Spinal Cord Injury*. 2018. https://www.hse.ie/eng/services/publications/clinical-strategy-and-programmes/integrated-care-pathway-spinal-cord-injury.pdf
38. Hentzen C, Biardeau X, Turmel N, et al. Prioritization of risk situations in neuro-urology: Guidelines from Association Française d'Urologie (AFU), Association Francophone Internationale des Groupes d'Animation de la Paraplégie (A.F.I.G.A.P.), Groupe de Neuro-urologie de Langue Française (GENULF), Société Française de Médecine Physique et de Réadaptation (SOFMER) and Société Interdisciplinaire Francophone d'UroDynamique et de Pelvi-Périnéologie (SIFUD-PP). *World J Urol*. 2022;40(1):133-139.
39. Ho C, Atchison K, Noonan VK, et al. Models of care delivery from rehabilitation to community for spinal cord injury: A scoping review. *J Neurotrauma*. 2021;38(6):677-697.
40. Hornby TG, Reisman DS, Ward IG, et al. Clinical practice guideline to improve locomotor function following chronic stroke, incomplete spinal cord injury, and brain injury. *J Neurol Phys Ther*. 2020;44(1):49-100.
41. Houghton PE, Campbell KE, Panel C. *Canadian Best Practice Guidelines for the Prevention and Management of Pressure Ulcers in People with Spinal Cord Injury*. Toronto: Ontario Neurotrauma Foundation; 2019.
42. Johns J, Krogh K, Rodriguez GM, et al. Management of neurogenic bowel dysfunction in adults after spinal cord injury: Clinical practice guideline for health care providers. *Top Spinal Cord Inj Rehabil*. 2021;27(2):75-151.
43. Jones ML, Gassaway J, Sweatman WM. Peer mentoring reduces unplanned readmissions and improves self-efficacy following inpatient rehabilitation for individuals with spinal cord injury. *J Spinal Cord Med*. 2021;44(3):383-391.
44. Kavanagh A, Baverstock R, Campeau L, et al. Canadian Urological Association guideline: Diagnosis, management, and surveillance of neurogenic lower urinary tract dysfunction. *Can Urol Assoc J*. 2019;13(6):E157-E176.
45. Kleemann S, Mosley I, Fitzgerald M. Mapping the continuum of care to surgery following traumatic spinal cord injury. *Injury*. 2018;49(8):1552-1557.
46. Kornhall DK, Jørgensen JJ, Brommeland T, et al. The Norwegian guidelines for the prehospital management of adult trauma patients with potential spinal injury. *Scand J Trauma Resusc Emerg Med*. 2017;25(1):2.
47. Krassioukov A, Biering-Sørensen F, Donovan W, et al. International standards to document remaining autonomic function after spinal cord injury. *J Spinal Cord Med*. 2012;35(4):201-10.
48. Krassioukov A, Linsenmeyer TA, Beck LA, et al. Evaluation and management of autonomic dysreflexia and other autonomic dysfunctions: Preventing the highs and lows: management of blood pressure, sweating, and temperature dysfunction. *Top Spinal Cord Inj Rehabil*. 2021;27(2):225-290.
49. Lala D, Houghton PE, Kras-Dupuis A, Wolfe DL. Developing a model of care for healing pressure ulcers with electrical stimulation therapy for persons with spinal cord injury. *Top Spinal Cord Inj Rehabil*. 2016;22(4):277-287.
50. Lamontagne ME, Best KL, Clarke T, Dumont FS, Noreau L. Implementation evaluation of an online peer-mentor training program for individuals with spinal cord injury. *Top Spinal Cord Inj Rehabil*. 2019;25(4):303-315.
51. Lee J, Milligan J, Hillier L, McMillan C. Evaluation of a primary care-based mobility clinic: improving health care for individuals with mobility impairments in Ontario, Canada. *Int J Disabil Community Rehabil*. 2014;13(1).
52. Li QP, Li J, Pan HY. Effects of Online home nursing care model application on patients with traumatic spinal cord injury. *Risk Manag Healthc Policy*. 2021;14:1703-1709.
53. Loh E, Mirkowski M, Agudelo AR, et al. The CanPain SCI clinical practice guidelines for rehabilitation management of neuropathic pain after spinal cord injury: 2021 update. *Spinal Cord*. 2022; 60:548-566.
54. Lukersmith S, Radbron L, Hopman K. Development of clinical guidelines for the prescription of a seated wheelchair or mobility scooter for people with traumatic brain injury or spinal cord injury. *Aust Occup Ther J*. 2013;60(6):378-386.
55. Madaris LL, Onyebueke M, Liebman J, Martin A. SCI hospital in home program: Bringing hospital care home for veterans with spinal cord injury. *Nurs Adm Q*. 2016;40(2):109-114.

56. Martin Ginis KA, van der Scheer JW, Latimer-Cheung AE, et al. Evidence-based scientific exercise guidelines for adults with spinal cord injury: An update and a new guideline. *Spinal Cord*. 2018;56(4):308-321.
57. Maschmann C, Jeppesen E, Rubin MA, Barfod C. New clinical guidelines on the spinal stabilisation of adult trauma patients - consensus and evidence based. *Scand J Trauma Resusc Emerg Med*. 2019;27(1):77.
58. McColl MA, Aiken A, McColl A, Sakakibara B, Smith K. Primary care of people with spinal cord injury: scoping review. *Can Fam Physician*. 2012;58(11):1207-16, e626-35.
59. McKim DA, Road J, Avendano M, et al. Home mechanical ventilation: A Canadian Thoracic Society clinical practice guideline. *Can Respir J*. 2011;18(4):197-215.
60. Mehta S, Guy SD, Bryce TN, et al. The CanPain SCI clinical practice guidelines for rehabilitation management of neuropathic pain after spinal cord: Screening and diagnosis recommendations. *Spinal Cord*. 2016;54(suppl 1):S7-s13.
61. Middleton JM, Sharwood LN, Cameron P, et al. Right care, right time, right place: improving outcomes for people with spinal cord injury through early access to intervention and improved access to specialised care: study protocol. *BMC Health Serv Res*. 2014;14:600.
62. Milligan J, Lee J. Enhancing primary care for persons with spinal cord injury: More than improving physical accessibility. *J Spinal Cord Med*. 2016;39(5):496-499.
63. Accident Compensation Corporation and the Ministry of Health. *New Zealand Spinal Cord Impairment Action Plan 2014-2019*. 2014. https://www.moh.govt.nz/notebook/nbbooks.nsf/0/BDD21D43769E4AD3CC257D08007EACF8/$file/spinal%20cord%20injury.pdf
64. Morse LR, Biering-Soerensen F, Carbone LD, et al. Bone mineral density testing in spinal cord injury: 2019 ISCD Official Position. *J Clin Densitom*. 2019;22(4):554-566.
65. Multidisciplinary Association of Spinal Cord Injury Professionals. *Clinical Guideline for Standing in Adults Following Spinal Cord Injury*. United Kingdom and Ireland: Spinal Cord Injury Centre Physiotherapy Lead Clinicians; 2013.
66. Nash MS, Groah SL, Gater DR, Jr., et al. Identification and management of cardiometabolic risk after spinal cord injury: Clinical practice guideline for health care providers. *Top Spinal Cord Inj Rehabil*. 2018;24(4):379-423.
67. Nash MS, Groah SL, Gater DR, et al. Identification and management of cardiometabolic risk after spinal cord injury. *J Spinal Cord Med*. 2019;42(5):643-677.
68. National Health Services. *Service Specifications: Spinal Cord Injury Services*. 2019. Accessed July 1, 2023. https://www.england.nhs.uk/wp-content/uploads/2019/04/service-spec-spinal-cord-injury-services-all-ages.pdf
69. Neurosurgery Educator and Outreach Network. *Provincial Guidelines for Spinal Cord Assessment*. Critical Care Services Ontario; 2016.
70. New South Wales Agency for Clinical Innovation. *Spinal Cord Injury Model of Care - Diagnostic Report*. 2017. https://aci.health.nsw.gov.au/networks/spinal-cord-injury
71. National Institute for Health and Care Excellence (NICE). *Neuropathic pain in adults: Pharmacological management in non-specialist settings*. London: NICE Clinical Guidelines; 2020.
72. National Clinical Guideline Centre. *Urinary Incontinence in Neurological Disease: Management of Lower Urinary Tract Dysfunction in Neurological Disease*. London: Royal College of Physicians (UK); 2012.
73. Nicolle LE, Gupta K, Bradley SF, et al. Clinical practice guideline for the management of asymptomatic bacteriuria: 2019 update by the Infectious Diseases Society of America. *Clin Infect Dis*. 2019;68(10):e83-e110.
74. Noonan VK, Soril L, Atkins D, et al. The application of operations research methodologies to the delivery of care model for traumatic spinal cord injury: The access to care and timing project. *J Neurotrauma*. 2012;29(13):2272-82.
75. Norton L, Parslow NJ, Johnston D, et al. *Best practice recommendations for the prevention and management of pressure injuries*. North York: Wounds Canada; 2017.
76. Nuwer MR, Emerson RG, Galloway G, et al. Evidence-based guideline update: intraoperative spinal monitoring with somatosensory and transcranial electrical motor evoked potentials: report of the Therapeutics and Technology Assessment Subcommittee of the American Academy of Neurology and the American Clinical Neurophysiology Society. *J Clin Neurophysiol*. 2012;29(1):101-108.
77. O'Dell L, Earle S, Rixon A, Davies A. Role of peer support for people with a spinal cord injury. *Nurs Stand*. 2019;34(4):69-75.
78. Osipowicz J, Nance M, Case A. *Emergency Department and PICU Clinical Pathway for Evaluation/Treatment of Children with Suspected Traumatic Spinal Cord Injury*. Children's Hospital of Philadelphia; 2023.
79. Osman A, Kumar N, Chowdhury J. The evolution of national care pathways in spinal cord injury management. *Trauma*. 2017;19(1 suppl):4-9.
80. Osmonov D, Christopher AN, Blecher GA, et al. Clinical recommendations from the European Society for Sexual Medicine exploring partner expectations, satisfaction in male and phalloplasty cohorts, the impact of penile length, girth and implant type, reservoir placement, and the influence of comorbidities and social circumstances. *J Sex Med*. 2020;17(2):210-237.
81. Picinich C, Madden LK, Brendle K. Activation to arrival: Transition and handoff from emergency medical services to emergency departments. *Nurs Clin North Am*. 2019;54(3):313-323.
82. Pilusa SI, Myezwa H, Potterton J. Services and interventions needed to prevent secondary health conditions throughout the life span of people with spinal cord injury, South Africa. *Afr J Disabil*. 2022;11:881.

83. Prange-Lasonder GB, Alt Murphy M, Lamers I, et al. European evidence-based recommendations for clinical assessment of upper limb in neurorehabilitation (CAULIN): Data synthesis from systematic reviews, clinical practice guidelines and expert consensus. *J Neuroeng Rehabil*. 2021;18(1):162.
84. Queensland Spinal Cord Injuries Service. *Queensland Spinal Cord Injuries Service Model of Care*. 2018. Accessed July 24, 2023. https://www.health.qld.gov.au/__data/assets/pdf_file/0025/424735/qscis-moc-2018.pdf
85. Quddusi A, Pedro KM, Alvi MA, Hejrati N, Fehlings MG. Early surgical intervention for acute spinal cord injury: Time is spine [published online July 19, 2023]. *Acta Neurochir (Wien)*.
86. Rapidi CA, Tederko P, Moslavac S, et al. Evidence-based position paper on Physical and Rehabilitation Medicine (PRM) professional practice for persons with spinal cord injury. The European PRM position (UEMS PRM Section). *Eur J Phys Rehabil Med*. 2018;54(5):797-807.
87. Roquilly A, Vigué B, Boutonnet M, et al. French recommendations for the management of patients with spinal cord injury or at risk of spinal cord injury. *Anaesth Crit Care Pain Med*. 2020;39(2):279-289.
88. Rudden L, Tolkin J. *Pediatric Spinal Cord Injury Clinical Pathway*. https://hollandbloorview.ca/sites/default/files/2019-06/Pediatric%20Rehabilitation%20Spinal%20Cord%20Injury%20%28SCI%29%20Personalized%20Clinical%20Pathway%20Draft%20IScos%202018.pdf
89. Ruediger M, Kupfer M, Leiby BE. Decreasing re-hospitalizations and emergency department visits in persons with recent spinal cord injuries using a specialized medical home. *J Spinal Cord Med*. 2021;44(2):221-228.
90. Russell HF, Richardson EJ, Bombardier CH, et al. Professional standards of practice for psychologists, social workers, and counselors in SCI rehabilitation. *J Spinal Cord Med*. 2016;39(2):127-45.
91. Ryken TC, Hadley MN, Aarabi B, et al. Management of acute combination fractures of the atlas and axis in adults. *Neurosurgery*. 2013;72(suppl 2):151-8.
92. Santos A, Gurling J, Dvorak MF, et al. Modeling the patient journey from injury to community reintegration for persons with acute traumatic spinal cord injury in a Canadian centre. *PLoS One*. 2013;8(8):e72552.
93. Schurch B, Iacovelli V, Averbeck MA, Stefano C, Altaweel W, Finazzi Agrò E. Urodynamics in patients with spinal cord injury: A clinical review and best practice paper by a working group of The International Continence Society Urodynamics Committee. *Neurourol Urodyn*. 2018;37(2):581-591.
94 Sethi RK, Wright AK, Nemani VM, et al. Team approach: Safety and value in the practice of complex adult spinal surgery. *JBJS Rev*. 2020;8(4):e0145.
95. Stansby G, Avital L, Jones K, Marsden G, Guideline Development G. Prevention and management of pressure ulcers in primary and secondary care: summary of NICE guidance. *BMJ*. 2014;348:g2592.
96. Stein DM, Knight WA 4th. Emergency neurological life support: Traumatic spine injury. *Neurocrit Care*. 2017;27(suppl 1):170-180.
97. Portelli Tremont JN, Cook N, Murray LH, Udekwu PO, Motameni AT. Acute traumatic spinal cord injury: Implementation of a multidisciplinary care pathway. *J Trauma Nurs*. 2022;29(4):218-224.
98. Tweedy SM, Beckman EM, Geraghty TJ, et al. Exercise and sports science Australia (ESSA) position statement on exercise and spinal cord injury. *J Sci Med Sport*. 2017;20(2):108-115.
99. Vaikuntam BP, Middleton JW, McElduff P, et al. Identifying predictors of higher acute care costs for patients with traumatic spinal cord injury and modeling acute care pathway redesign: A record linkage study. *Spine (Phila Pa 1976)*. 2019;44(16):E974-E983.
100. Vaikuntam BP, Middleton JW, McElduff P, et al. Assessing the impact of care pathways on potentially preventable complications and costs for spinal trauma patients: protocol for a data linkage study using cohort study and administrative data. *BMJ Open*. 2018;8(11):e023785.
101. van de Pol E, Lucas K, Geraghty T, et al. The delivery of specialist spinal cord injury services in Queensland and the potential for telehealth. *BMC Health Serv Res*. 2016;16:29.
102. van der Scheer JW, Goosey-Tolfrey VL, Valentino SE, Davis GM, Ho CH. Functional electrical stimulation cycling exercise after spinal cord injury: A systematic review of health and fitness-related outcomes. *J Neuroeng Rehabil*. 2021;18(1):99.
103. Walia S, Wolfe D, Keast D, et al. Facilitators and barriers for implementing an internet clinic for the treatment of pressure injuries. *Telemed J E Health*. 2019;25(12):1237-1243.
104. Walters BC, Hadley MN, Hurlbert RJ, et al. Guidelines for the management of acute cervical spine and spinal cord injuries: 2013 update. *Neurosurgery*. 2013;60(CN_suppl_1):82-91.
105. Yli-Hankala A, Chew MS, Olkkola KT, Rehn M, Sverrisson K, Møller MH. Clinical practice guideline on spinal stabilisation of adult trauma patients: Endorsement by the Scandinavian Society of Anaesthesiology and Intensive Care Medicine. *Acta Anaesthesiol Scand*. 2021;65(7):986-987.
106. Yue JK, Chan AK, Winkler EA, Upadhyayula PS, Readdy WJ, Dhall SS. A review and update on the guidelines for the acute management of cervical spinal cord injury - Part II. *J Neurosurg Sci*. 2016;60(3):367-384.
107. Zehr EP. Evidence-based risk assessment and recommendations for physical activity clearance: Stroke and spinal cord injury. *Appl Physiol Nutr Metab*. 2011;36(suppl 1):S214-31.
