# Supplementary material for: What Is the Pathway to the Best Model of Care for Traumatic Spinal Cord Injury? Evidence-Based Guidance
Source: Top Spinal Cord Inj Rehabil. 2023 Nov 17;29(Suppl):103–11. doi: 10.46292/sci23-00059S (PMC10759857; doi:10.46292/sci23-00059S)
Supplement: Supplementary file 2 [file i1945-5763-29-suppl-103_s02.pdf]

eAPPENDIX 2

eTable 1. Outcomes of the regional consultations about gaps and priorities of the tSCI Care Pathway. Ontario Health Region: Central

Priority	Improve education/training for healthcare providers	Improve education/training for personal support workers
**Solutions**	Environmental scan to identify what type of education specialized and nonspecialized healthcare providers receive, what current specialized education programs exist, and potential interests for a wide range of healthcare providers.	Develop and implement a regional and provincial environmental scan to identify what type of basic education PSWs receive and if there is specific tSCI content – what current PSW education programs exist and their curriculum.
	Create an educational program to share knowledge between specialized and nonspecialized healthcare care providers.	Create a consensus process to standardize PSW training across the province. This could include other attendant care groups/organizations that worked on standardizing training.
	Identify mechanisms to implement the Accessibility for Ontarians with Disabilities Act (AODA is mandatory by 2025).	Using the outcomes of activities 1 and 2, create an advocacy model to influence change in basic education for PSWs, including basic tSCI training during PSW education, and offer a specialized tSCI module that follows the basic training.
	Environmental scan to identify existing mechanisms to link specialized and nonspecialized healthcare providers in the hospital and in the community for educational purposes.	Improve education/training for personal support workers.
	Improve the awareness of specialized training for nonregulated healthcare professionals (e.g., personal support workers).	

Note: PSW = personal support worker; tSCI = traumatic spinal cord injury.

This supplementary digital material accompanies
Top Spinal Cord Inj Rehabil 2023;29(suppl):103-111
© 2023 American Spinal Injury Association
www.asia-spinalinjury.org
doi: 10.46292/sci23-00059S

eTable 2. Outcomes of the regional consultations about gaps and priorities of the tSCI Care Pathway. Ontario Health Region: East

**Priority**	**Improve access to community services and supports**	**Improve education/training for healthcare providers**
**Solutions**	Environmental scan about the current engagement of the healthcare system with community organizations providing services and supports.	Environmental scan for tSCI-specific education programs for different tSCI care providers; create a centralized virtual platform to share the educational programs identified (including content update mechanism).
	Environmental scan about current specialized navigation roles in other injuries (e.g., brain injury navigators) and how this role could be adapted to tSCI.	Explore the structure of existing peer mentorship planforms for healthcare providers in other conditions and use this knowledge to create a tSCI-specific mentorship platform. Include a strong method to engage nonspecialized tSCI healthcare providers.
	Build new or update existing care maps that include specialized system navigation; include system navigation in the accreditation standards; standardize quality indicators for transitions to community.	Quality indicators currently exist to address existing and expected tSCI-specific education for specialized rehab providers (i.e., SCI Consortium). These could be expanded to other stages of care and care environments.

Note: tSCI = traumatic spinal cord injury.

eTable 3. Outcomes of the regional consultations about gaps and priorities of the tSCI Care Pathway. Ontario Health Region: North East

**Priority**	**Improve education/training for healthcare providers**	**Improve care coordination**
**Solutions**	Create awareness materials and ongoing educational sessions (e.g., webinars, rounds, corporate training) of available tSCI-specific educational programs for different healthcare providers. SCIO has an existing free platform (e.g., Cortree Disability Education Centre) and Can-SCIP could be leveraged.	Create care maps that account for patient/injury-specific needs and available hospital resources (allied health, knowledge/skills, infrastructure, specialized clinics/staff) to facilitate the decision-making process related to referring patients to specialized vs. nonspecialized hospitals. Build list of resources/inventory into care maps and share it with case managers for appropriate linkages.
	Evaluate the current state of mandatory corporate training for tSCI for new hires and update them as needed (could be merged with SCIO). Explore the option of making this training ongoing (e.g., yearly refreshers).	Create awareness within the healthcare system about how/when to engage SCIO and the services they provide.
	Create a support network (peer mentorship program) to connect nonspecialized North East healthcare providers with specialized centres. and networks (e.g., SCIO, Spinal Cord Injury Implementation & Evaluation Quality Care Consortium, best practice rounds from specialized centres).	Improve the awareness of Home and Community Care Support Services about the services provided by SCIO and vice versa.
		Explore the available mechanism to support transportation services in the region (e.g., Priority Assistance to Transition Home program – red cross; Platinum services – private and public) and explore the possibility of expanding existing programs and improving awareness to healthcare providers and persons with lived experience. This considers transportation for the person with lived experience and family/friends or support personnel (e.g., case managers, nurses, personal support workers, therapists).

Note: SCIO = Spinal Cord Injury Ontario; tSCI = traumatic spinal cord injury.

eTable 4. Outcomes of the regional consultations about gaps and priorities of the tSCI Care Pathway. Ontario Health Region: North West

Priority	Improve education/training for healthcare providers	Increase available tSCI-specific housing
**Solutions**	Explore availability of community services and supports in the region and province to inform tSCI-specific educational programs for healthcare providers. SCIO has educational programs (Cortree Disability Education Centre).	Identify existing infrastructure available (empty hotels, long-term care, supportive housing) to be renovated to become housing for persons living with tSCI and make partnerships to secure long-term funding.
	Explore opportunities to integrate educational efforts of community regional associations and networks to create integrated tSCI-specific education programs for healthcare providers.	Increase capacity (i.e., funding to hire and retain staff, specialized, training, remove barriers to access staff funding when the housing space is available) for existing community supports (i.e., North West | Home and Community Care Support Services; also Community Services for Independence North West)
	Create a model for tSCI specialized education specific to all involved in tSCI care (regulated and nonregulated professionals) that includes: educational program delivered by specialists and persons with lived experience; a process to facilitate Q&A during and after the educational program (e.g., peer mentorship model); and incentives to attend and complete programs.	Strengthen the engagement with other organizations and associations to improve advocacy efforts to increase access to supported housing (from a funding perspective).
	Create a mechanism to support advocacy efforts to restart certification programs for rehabilitation nurses.	Increase funding and specialized advice for those needing housing renovations or home supports.

Note: SCIO = Spinal Cord Injury Ontario; tSCI = traumatic spinal cord injury.

eTable 5. Outcomes of the regional consultations about gaps and priorities of the tSCI Care Pathway. Ontario Health Region: Toronto

**Priority**	**Improve access to specialized personal support workers in hospitals and the community**	**Improve access to primary care**
**Solutions**	Perform an environmental scan to better understand: (a) available education/training/certification for PSWs, from provincial and regional perspectives and healthcare funding sources; (b) current gaps of PSW services in hospitals and the community (e.g., own home, residential facilities, long-term care), from provincial and regional perspectives and healthcare funding source.	Implement an environmental scan to identify primary care perceptions and barriers in accepting individuals with complex cases (tSCI).
	Perform surveys with people with lived experience and their family and friends to identify needs and preferences to inform educational programs for PSWs. This survey could be performed every time PSW educational programs are updated.	Implement an environmental scan to identify funding (e.g., training, equipment, office improvements) and networks of support (including connections with specialized providers and care coordination supports) for primary care attending individuals with complex cases (e.g., tSCI with concurrent mental illness).
	Use the outcomes of the environmental scan and surveys to drive changes in or expand the reach of existing education, training, and certification programs for PSWs.	Use the outcomes of the environmental scans to drive calls for action to target improving uptake of complex cases by primary care providers.
	Create a consensus process to define the roles and responsibilities of PSWs to work with tSCI.	

Note: PSW = personal support worker; tSCI = traumatic spinal cord injury.

eTable 6. Outcomes of the regional consultations about gaps and priorities of the tSCI Care Pathway. Ontario Health Region: West

**Priority**	**Improve care and service coordination**	**Improve access and affordability of specialized healthcare/services in the community**
**Solutions**	Perform an environmental scan to list intake criteria for different hospitals (specialized or nonspecialized), rehab centres (specialized and nonspecialized), tSCI-specific community services and supports (e.g., housing, OTs, PTs, attendant services, nursing, specialized services), funding source (public vs. private).	Redesign referral pathways and intake criteria to specialized inpatient rehab (e.g., patient-specific goals) after acute care to adequately connected to spec outpatient services (wound care, wheelchair prescription/adjustments, sexual health), team-based primary care providers (e.g., family health teams), community supports (SCIO), and specialized community rehab (e.g., physio, pain, physical fitness program).
	Explore the possibility of establishing a common language to appropriately categorize patients so that their care needs are understood, and appropriate care coordination is implemented. (i.e., stroke could serve as a model.	Create business plans to support the expansion of team-based primary care teams (e.g., family health teams) to provide care for people with lived experience, family, and friends and to support nonspecialized professionals attending tSCI patients.
	Explore the possibility of creating a specialized system navigation role for tSCI. This position could focus on healthcare education, care coordination, and advocating for staff and patients. Part of this project would involve comparing the brain injury and stroke navigation role with the services provided by SCIO. Next steps would include piloting and broad implementation across the province.	Physiatry E-consult model to support primary care physicians in effectively managing secondary complications of their tSCI patients (pathway already exists in primary care for other specialties, e.g., geriatric consults).
	Explore the processes that different regional hospitals use to implement patient transitions across different levels of the healthcare system.	

Note: SCIO = Spinal Cord Injury Ontario; tSCI = traumatic spinal cord injury.
